# Metabolic syndrome is a risk factor for cancer mortality in the general Japanese population: the Jichi Medical School Cohort Study

**DOI:** 10.1186/s13098-018-0398-2

**Published:** 2019-01-09

**Authors:** Jun Watanabe, Eiichi Kakehi, Kazuhiko Kotani, Kazunori Kayaba, Yosikazu Nakamura, Shizukiyo Ishikawa

**Affiliations:** 1 Division of Community and Family Medicine, Center for Community Medicine, Jichi Medical University, 3311-1 Yakushiji, Shimotsuke, Tochigi 329-0498 Japan; 2 Department of General Medicine, Tottori Municipal Hospital, 1-1 Matoba, Tottori, Tottori Japan; 3 Graduate School of Saitama Prefectural University, 820 Sannomiya, Koshigaya, Saitama Japan

**Keywords:** Metabolic syndrome, Cohort studies, Neoplasm, Mortality, Japanese

## Abstract

**Background:**

Metabolic syndrome (MetS) and cancer are major public health problems worldwide. The relationship between MetS and cancer death is of great interest. We examined the predictive value of MetS for cancer mortality in Japan.

**Methods:**

Study participants included 4495 men and 7028 women aged 18–90 years who were registered between 1992 and 1995 as part of the Jichi Medical School Cohort Study. We used a definition of MetS modified for the Japanese population. The primary outcome was cancer mortality. Additionally, the relationship between MetS and cancer-type specific mortality was examined. Analyses were conducted with Cox’s regression models adjusted for age, smoking status, alcohol drinking status, marital status, educational attainment, physical activity, occupational category, and menopausal status (only in women).

**Results:**

During a mean follow-up of 18.5 years, 473 men and 297 women died from cancer. MetS was positively associated with cancer mortality in women (hazard ratio [HR], 1.69; 95% confidence interval [CI] 1.21–2.36), but not in men (HR, 1.21; 95% CI 0.90–1.62). Additionally, MetS was associated with a high risk of colorectal (HR, 3.48; 95% CI 1.68–7.22) and breast (HR, 11.90; 95% CI 2.25–62.84) cancer deaths in women.

**Conclusion:**

MetS was a significant predictor of cancer mortality in women.

**Electronic supplementary material:**

The online version of this article (10.1186/s13098-018-0398-2) contains supplementary material, which is available to authorized users.

## Background

Metabolic syndrome (MetS) is a disease characterized by a cluster of high blood glucose, dyslipidemia, obesity, and hypertension [1]. MetS is an important risk factor for not only cardiovascular diseases (CVD) but also the development of cancer [2, 3]. Accumulating evidence regarding the clinical value of MetS in estimating the risk of cancer has led to increased interest in the relationship between MetS and cancer.

Cancer remains a major cause of death worldwide, with 14.1 million new cases and 8.2 million deaths from cancer occurring annually [4]. Of note, cancer deaths in Japan have been gradually increasing and now constitute the leading cause of death in the country [5]. Each component of MetS, viz., obesity [6], hypertension [7], hyperglycemia [8–11], and dyslipidemia [12], independently increases the risk of cancer. However, it remains unclear whether there is a dose–response association between MetS components and cancer mortality. Despite substantial interest in the relationship between MetS and cancer deaths, few studies have examined the contribution of the syndrome to cancer deaths [13–17].

We herein investigated the relationship between MetS and cancer mortality in a general Japanese population.

## Methods

### Participants and follow-up

The present study was a serial prospective population-based cohort analysis using data from the Jichi Medical School (JMS) Cohort Study. The research design of the JMS Cohort Study and some descriptive data have been reported in detail elsewhere [18]. The study was initiated in 1992 to investigate the relationship between potential risk factors and CVD in the general Japanese population. Baseline data in 12 Japanese communities were obtained between April 1992 and July 1995 from national mass screening examinations for CVD, which were conducted according to the Health and Medical Service Law for the Aged in Japan. Local municipal governments sent personal invitations through the mail to all mass screening participants. Participants were aged 40–69 years in eight areas (Iwaizumi, Tako, Kuze, Sakuma, Sakugi, Okawa, Ainoshima, and Akaike), ≥ 35 years in one area (Wara), and ≥ 18 years in three areas (Hokudan, Yamato, and Takasu). We included 12,490 participants (4911 men and 7579 women), and the follow-up rate was 99% in 18 years from the data of baseline registration to the end of 2013. After the exclusion of 889 participants, including those who were lost to follow-up (n = 97), had a history of cancer (n = 141), had missing data for height and weight (n = 494) or blood pressure and blood samples (n = 157), or died in the first 2 years of follow-up (n = 78), the remaining 11,523 participants (4495 men and 7028 women) were eligible for the analysis.

The JMS Cohort Study conducted follow-up surveys until 31 December 2013. We obtained death certificates from public health centers with the permission of the Agency of General Affairs and the Ministry of Health, Labour and Welfare. Each municipal government collected annual data on participant relocation. In Japan, the registries of residency and deaths are established by law and doctors are trained to describe in standard format.

### Measurements and outcomes

In the baseline survey, height without shoes and weight of clothed participants were measured, and body mass index (BMI) was calculated as weight (kg)/height (m)^2^. Trained interviewers used a standardized questionnaire to obtain data, including smoking habit (never, past, or current smoker), alcohol drinking habit (never, past, or current drinker), medical history (past or present hypertension, diabetes, and hyperlipidemia, and the presence of these medication), marital status (yes or no), educational attainment (the age at completion of education), physical activity (the Framingham Study Questionnaire [19]), occupation, and menopause status (pre or post) in women. Educational attainment was categorized into less than junior high school (≤ 15 years), high school (16–18 years), and more than high school (≥ 19 years). Physical activity was categorized by using physical activity index (PAI) estimated by calculating the coefficients and time spent on an activity, into low (PAI < 30), middle (PAI = 30–39), and high (PAI ≥ 40) in this study [19–21]. Occupation was categorized into white-collar, blue-collar, or no working. Sales workers, clerks, professional/technicians, and service workers were categorized as white-collar occupations. Agriculture and forestry, fishery, security, transportation/communications, civil engineering and construction, and craft workers/laborers were categorized as blue-collar occupations, while retiree and inoccupation were categorized as no working [22]. Systolic blood pressure (SBP) and diastolic blood pressure (DBP) were measured using a fully automated sphygmomanometer, BP203RV-II (Nippon Colin Co., Ltd., Komaki, Japan), on the right arm in the sitting position after at least 5 min of rest. Serum total cholesterol (TC), high-density lipoprotein cholesterol (HDL-C), triglyceride (TG), and plasma glucose (PG) concentrations were measured using enzymatic methods, as previously reported [18].

Information on the causes of death were determined by death certificates and coded using the International Classification of Diseases 10th revision (ICD-10). The primary endpoint was total cancer deaths (C00–C97), and secondary outcomes were lung (C33–34), stomach (C16), colon (C18), rectum (C19–20), liver (C22), gallbladder (C23), prostate (C61), and breast (C50) cancer deaths.

### Definition

We applied the modified Japanese MetS definition using BMI instead of waist circumference (WC) because only approximately 20% of all participants in the JMS Cohort Study had WC measured, and BMI ≥ 25 kg/m^2^ is consistent with a WC of ≥ 85 cm in men and ≥ 90 cm in women in Japan [23]. MetS was defined as BMI ≥ 25 kg/m^2^ and the presence of two or more of the following: (i) SBP and/or DBP ≥ 130/85 mmHg or the use of antihypertensive medication; (ii) TG ≥ 1.69 mmol/L (150 mg/dL) and/or HDL-C < 1.03 mmol/L (40 mg/dL) and/or the use of antihyperlipidemic medication; and (iii) fasting PG ≥ 6.1 mmol/L (110 mg/dL) (with a fasting duration of at least 3 h) or casual PG (for less than 3 h or without regard to the time since the last meal) ≥ 7.8 mmol/L (140 mg/dL) and/or the use of antidiabetic medication.

### Statistical analysis

Summary statistics were used to compare the characteristics of participants with and without MetS using the Mann–Whitney U test and χ^2^ test. To elucidate the relationship between MetS and cancer mortality, a Cox’s proportional hazards regression model was constructed to estimate multivariate-adjusted hazard ratios (HRs) and 95% confidence intervals (CIs) for cancer mortality to the number of metabolic risk factors, the obesity category (BMI ≥ 25 kg/m^2^ or < 25 kg/m^2^), and MetS by sex, adjusting for age, smoking status (never, past, or current smoker), alcohol drinking status (never, past, or current drinker), marital status (yes or no), educational attainment (≤ 15, 16–18, or ≥ 19 years), physical activity (low, middle, high), occupation category (white-collar, blue-collar, or no working), and menopausal status (pre or post) only in women at baseline. These covariates are commonly adjusted for in cancer risk. However, tests for linear trend across the number of metabolic risk factors were conducted by including an ordinal scoring in the models to examine a dose–response association between MetS component and cancer mortality. The proportional hazards assumption for the model was checked by examining the log-negative-log plot of the survival function for participants with and without MetS, and the number of MetS components against time to death/follow-up time. These curves help in identifying non-proportionality patterns in hazard function such as crossing of the curves, convergent, or divergent. Additionally, we conducted Cox’s regression analysis by age ≥ 65 years or < 65 years and estimated multivariate adjusted HRs for cancer mortality to each metabolic risk factor, and for cancer-type specific mortality associated with MetS by sex. We performed sensitivity analyses by excluding participants who were younger than 40 years at baseline to minimize the influence of a younger generation. The threshold for significance was P < 0.05. All statistical analyses were conducted using IBM SPSS version 25.0 (IBM Corp., Armonk, NY, USA).

## Results

The baseline characteristics of subjects with and without MetS are summarized for both the sexes in Table 1. The mean follow-up period was 18.5 (standard deviation [SD], 4.6) years. The median age of participants was 58 (interquartile range [IQR], 46–64) years in men and 57 (IQR, 47–64) years in women, and 91.3% of participants were older than 40 years. At baseline, 11.6% of men and 8.9% of women had MetS. There were no significant differences in smoking in men and women and alcohol drinking in men between participants with and without MetS. Both men and women with MetS had higher BMI, SBP, DBP, PG, TC and TG levels and lower HDL-C levels, compared to without MetS.

**Table 1:** Baseline characteristics of participants with or without metabolic syndrome by sex

	Men				P^a^	Women				P^a^
	Without MetS		With MetS			Without MetS		With MetS		
	N	%	N	%		N	%	N	%	
Number of participants	3973	88.4	522	11.6		6406	91.1	622	8.9	

	N	Median (IQR)	N	Median (IQR)	P^a^	N	Median (IQR)	N	Median (IQR)	P^a^
Age (year)		58 (45–64)		56 (46–63)	0.016		57 (47–64)		60 (53–65)	< 0.001
BMI (kg/m^2^)		22.4 (20.8–24.0)		26.6 (25.8–28.1)	< 0.001		22.6 (20.8–24.4)		27.0 (25.9–28.9)	< 0.001
SBP (mmHg)		128 (115–141)		144 (135–156)	< 0.001		124 (112–139)		143 (134–156)	< 0.001
DBP (mmHg)		77 (70–86)		88 (82–94)	< 0.001		75 (67–83)		86 (80–92)	< 0.001
Plasma Glucose (mmol/L)		5.4 (4.9–6.1)		6.0 (5.3–7.1)	< 0.001		5.3 (4.9–5.8)		6.0 (5.2–7.1)	< 0.001
Total cholesterol (mmol/L)		4.7 (4.2–5.3)		5.1 (4.4–5.7)	< 0.001		5.0 (4.4–5.6)		5.4 (4.8–6.0)	< 0.001
HDL-cholesterol (mmol/L)		1.2 (1.1–1.5)		1.0 (0.9–1.2)	< 0.001		1.3 (1.2–1.6)		1.1 (1.0–1.3)	< 0.001
Triglyceride (mmol/L)		1.1 (0.8–1.6)		2.1 (1.5–2.8)	< 0.001		1.0 (0.7–1.4)		1.9 (1.4–2.5)	< 0.001
Smoking		%		%			%		%	
Current	1946	49.0	230	44.1	0.101	336	5.3	30	4.8	0.345
Former	1062	26.7	149	28.5		174	2.7	11	1.8	
Never	804	20.2	120	23.0		5535	86.4	541	87.1	
Data missing	161	4.1	23	4.4		361	5.6	40	6.3	
Alcohol drinking										
Current	2811	70.8	343	65.7	0.210	1488	23.2	120	19.3	0.022
Former	123	3.1	22	4.2		78	1.2	14	2.2	
Never	788	19.8	107	20.5		4353	68.0	411	66.1	
Data missing	251	6.3	50	9.6		487	7.6	77	12.4	
Diabetes mellitus	83	2.1	22	4.2	0.002	67	1.0	43	6.9	< 0.001
Hypertension	334	8.4	99	19.0	< 0.001	619	9.7	205	33.0	< 0.001
Hyperlipidemia	43	1.1	11	2.1	0.002	93	1.5	49	7.9	< 0.001
Marital status										
Married	3644	91.7	473	90.6	0.278	5867	91.6	570	91.6	0.957
Single	310	7.8	48	9.2		519	8.1	50	8.0	
Data missing	19	0.5	1	0.2		20	0.3	2	0.3	
Education										
≤ 15 years	1766	44.5	224	42.9	0.730	3176	49.6	364	58.5	< 0.001
16–18 years	1683	42.4	231	44.3		2558	39.9	213	34.2	
≥ 19 years	503	12.7	67	12.8		650	10.1	43	6.9	
Data missing	21	0.5	0	0		22	0.3	2	0.3	
Physical activity										
Low	1232	31.0	204	39.1	0.001	2813	43.9	276	44.4	0.321
Middle	1824	45.9	211	40.4		2942	45.9	293	47.1	
High	730	18.4	81	15.5		335	5.2	24	3.9	
Data missing	187	4.7	26	5.0		316	4.9	29	4.7	
Occupation										
White-collar	869	21.9	142	27.2	0.002	1491	23.3	125	20.1	< 0.001
Blue-collar	2548	64.1	296	56.7		2296	35.8	192	30.9	
No working	532	13.4	84	16.1		2596	40.5	303	48.7	
Data missing	24	0.6	0	0		23	0.4	2	0.3	

*MetS* metabolic syndrome, *BMI* body mass index, *SBP* systolic blood pressure, *DBP* diastolic blood pressure, *HDL* high-density lipoprotein, *IQR* interquartile range

^a^The Mann–Whitney U test or χ^2^ test were performed

**Fig. 1:**
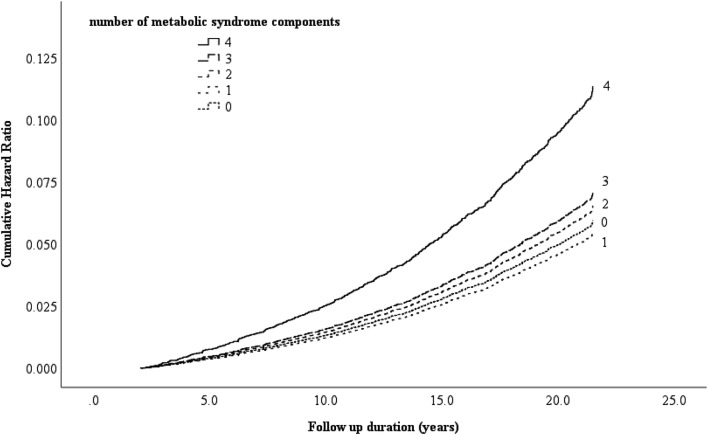
Adjusted hazard curves of cancer mortality with the number of metabolic syndrome components

Figure 1 shows adjusted hazard curves of cancer mortality with the number of MetS components by Cox regression analysis. The proportional hazards assumption for the model was reasonable because the log-negative-log plot showed the separate lines did not cross and were not convergent, and divergent in Additional file 1: Figure S1. Table 2 shows HRs and 95% CIs for cancer mortality with the number of Japanese MetS components and obesity category. Increases in the number of Japanese MetS components showed a linear association with the HRs for cancer mortality (P for trend = 0.007), especially in women (P for trend = 0.027), but not in men (P for trend = 0.10). The effects of obesity with 2–3 metabolic risk factors were significantly greater than those in participants who were not obese and had no risk factors, whereas the effects of not being obese but having 2–3 risk factors were not, especially in women.

**Table 2:** Multivariate analysis of cancer mortality with the number of metabolic syndrome components

	Participants	Cancer deaths	Person-years	Crude mortality (/1000 person-years)	Total HR-age^b^ (95% CI)	Total HR-all^c^ (95% CI)	Men HR-all^c^ (95% CI)	Women HR-all^c^ (95% CI)
No. of metabolic risk factors^a^								
0	3439	173	64,584	2.7	1.00	1.00	1.00	1.00
1	4111	269	75,684	3.6	1.02 (0.84–1.24)	0.92 (0.76–1.12)	0.95 (0.73–1.23)	0.88 (0.64–1.20)
2	2628	212	48,329	4.4	1.17 (0.95–1.43)	1.10 (0.89–1.36)	1.14 (0.87–1.49)	1.04 (0.74–1.46)
3	1149	90	21,050	4.3	1.23 (0.95–1.59)	1.19 (0.92–1.55)	1.08 (0.76–1.54)	1.37 (0.92–2.05)
4	196	26	3512	7.4	2.14 (1.41–3.23)	1.91 (1.23–2.96)	1.68 (0.95–2.95)	2.32 (1.16–4.66)
P for trend					0.002	0.007	0.103	0.027
Combination of obesity and 3 other risk factors								
Non-obesity and 0–1 risk factors	7077	422	131,208	3.2	1.00	1.00	1.00	1.00
Non-obesity and 2 risk factors	1451	136	26,162	5.2	1.25 (1.03–1.52)	1.18 (0.97–1.45)	1.28 (1.02–1.62)	0.89 (0.60–1.34)
Non-obesity and 3 risk factors	201	15	3499	4.3	1.04 (0.62–1.74)	0.98 (0.57–1.67)	0.99 (0.53–1.88)	0.90 (0.34–2.45)
Obesity and 0–1 risk factors	1650	96	31,268	3.1	0.93 (0.74–1.16)	1.04 (0.82–1.32)	0.85 (0.58–1.22)	1.28 (0.93–1.76)
Obesity and 2 risk factors	948	75	17,560	4.3	1.24 (0.97–1.59)	1.32 (1.02–1.71)	1.14 (0.80–1.61)	1.61 (1.10–2.36)
Obesity and 3 risk factors	196	26	3512	7.4	2.08 (1.40–3.10)	1.99 (1.31–3.04)	1.71 (0.99–2.95)	2.50 (1.27–4.92)

*HR* hazard ratio, *CI* confidence interval

^a^The 4 components of being obesity, having an elevated blood pressure, elevated plasma glucose, and dyslipidemia

^b^Hazard ratios adjusted for age

^c^Hazard ratios adjusted for age, smoking status (never, past, or current smoker), alcohol drinking status (never, past, or current drinker), marital status (yes or no), educational attainment (≤ 15, 16–18, or ≥ 19 years), physical activity (low, middle, high), occupation category (white-collar, blue-collar, or no working), and menopausal status (pre or post) only in women

**Fig. 2:**
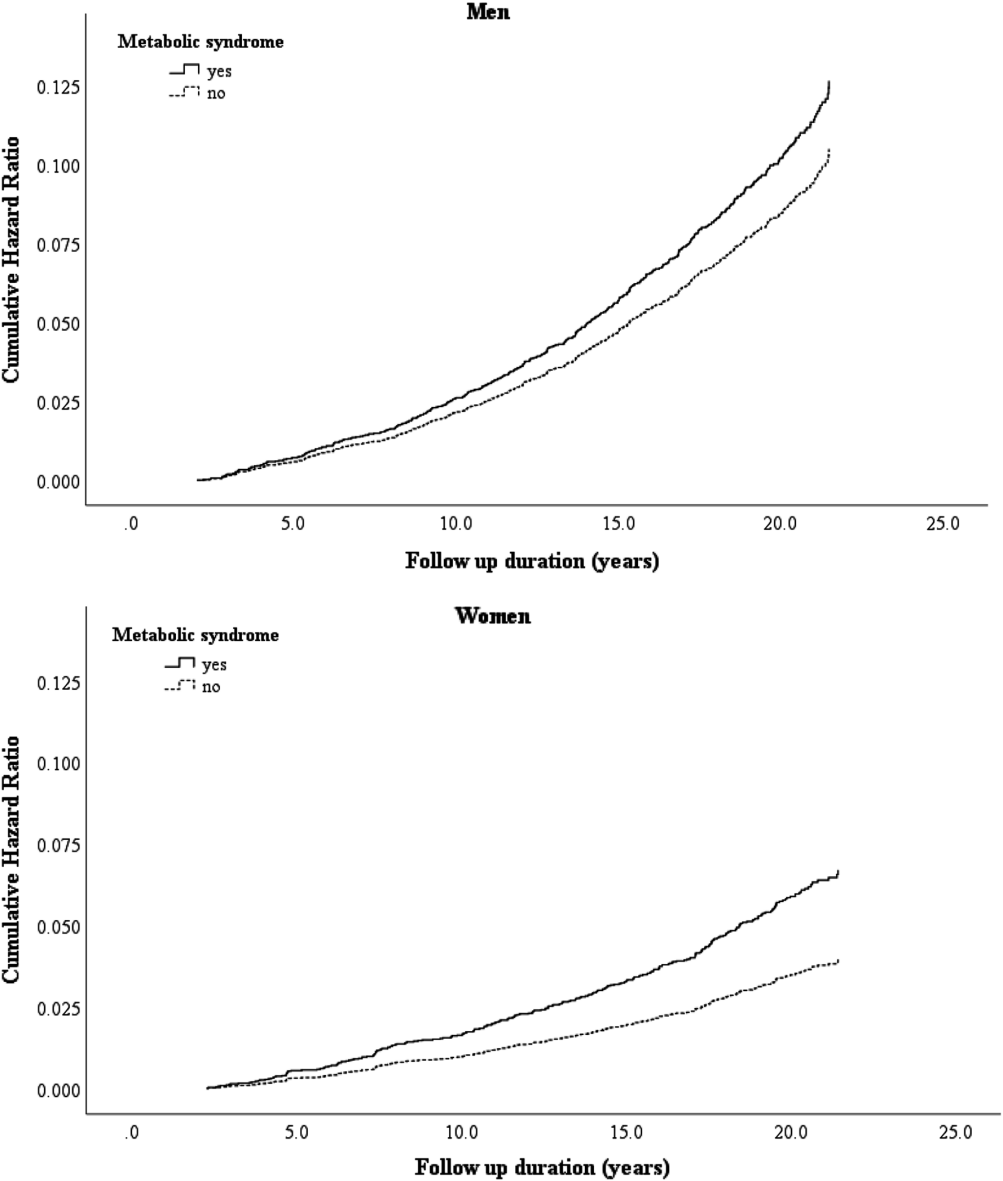
Adjusted hazard curves of cancer mortality with metabolic syndrome by sex

Figure 2 shows adjusted hazard curves of cancer mortality with metabolic syndrome by Cox regression analysis. The proportional hazard assumption for the model was reasonable in Additional file 1: Figure S2. Table 3 shows the number of deaths, crude mortality rates, and adjusted HRs for cancer mortality by sex. During the follow-up period, 770 deaths due to cancer (473 men and 297 women) occurred. Age-adjusted HRs were 1.11 (95% CI 0.84–1.48) in men and 1.69 (95% CI 1.23–2.31) in women. Multivariate-adjusted HRs were 1.21 (95% CI 0.90–1.62) in men and 1.69 (95% CI 1.21–2.36) in women. In addition, among women younger than 65 years, MetS was associated with a significantly increased risk of cancer mortality (multivariate-adjusted HR 1.66; 95% CI 1.09–2.55), whereas among women older than 65 years, there was no relationship between MetS and cancer mortality (multivariate-adjusted HR 1.69; 95% CI 0.99–2.89).

**Table 3:** Multivariate analysis of cancer mortality with metabolic syndrome by sex

	Men		Women	
	Without MetS	With MetS	Without MetS	With MetS
MetS participants, n (%)	3973 (88.4)	522 (11.6)	6406 (91.1)	622 (8.9)
Cancer deaths	418	55	251	46
Parson-Years	71,444	9418	120,718	11,654
Cancer mortality				
Crude mortality (/1000 person-years)	5.9	5.8	2.1	3.9
HR-Age^a^ (95% CI)	1.0 (reference)	1.11 (0.84–1.48)	1.0 (reference)	1.69 (1.23–2.31)
HR-All^b^ (95% CI)	1.0 (reference)	1.21 (0.90–1.62)	1.0 (reference)	1.69 (1.21–2.36)
< 65 years old				
Crude mortality (/1000 person-years)	3.3	3.8	1.3	2.4
HR-Age^a^ (95% CI)	1.0 (reference)	1.14 (0.80–1.61)	1.0 (reference)	1.70 (1.14–2.55)
HR-All^b^ (95% CI)	1.0 (reference)	1.22 (0.84–1.77)	1.0 (reference)	1.66 (1.09–2.55)
≥ 65 years old				
Crude mortality (/1000 person-years)	2.6	2.0	0.8	1.5
HR-Age^a^ (95% CI)	1.0 (reference)	1.09 (0.68–1.74)	1.0 (reference)	1.71 (1.03–2.83)
HR-All^b^ (95% CI)	1.0 (reference)	1.19 (0.73–1.95)	1.0 (reference)	1.69 (0.99–2.89)

*MetS* metabolic syndrome, *HR* hazard ratio, *CI* confidence interval

^a^Hazard ratios adjusted for age

^b^Hazard ratios adjusted for age, smoking status (never, past, or current smoker), alcohol drinking status (never, past, or current drinker), marital status (yes or no), educational attainment (≤ 15, 16–18, or ≥ 19 years), physical activity (low, middle, high), occupation category (white-collar, blue-collar, or no working), and menopausal status (pre or post) only in women

Table 4 shows the predictive effect of each MetS component on cancer mortality. The effects of obesity in women (multivariate-adjusted HR 1.48; 95% CI 1.15–1.91) and elevated PG in both men (multivariate-adjusted HR 1.49; 95% CI 1.18–1.88) and women (multivariate-adjusted HR 1.44; 95% CI 1.03–2.03) on predicting cancer mortality were significantly greater in participants with MetS than in those without the syndrome.

**Table 4:** Multivariate analysis of cancer mortality with metabolic syndrome according to each metabolic risk factor by sex

	Presence	Participants	Cancer deaths	Parson-years	Crude mortality (/1000 person-years)	HR-age^a^ (95% CI)	HR-all^b^ (95% CI)
Men							
Obesity	No	3484	378	62,224	6.1	1.00	1.00
	Yes	1011	95	18,602	5.1	0.97 (0.77–1.21)	0.99 (0.78–1.26)
Elevated blood pressure	No	2053	191	37,693	5.1	1.00	1.00
	Yes	2442	282	43,150	6.5	0.96 (0.80–1.16)	0.99 (0.82–1.21)
Elevated plasma glucose	No	3833	378	69,607	5.4	1.00	1.00
	Yes	662	95	11,247	8.4	1.46 (1.17–1.83)	1.49 (1.18–1.88)
Dyslipidemia	No	2676	280	48,221	5.8	1.00	1.00
	Yes	1819	193	32,633	5.9	1.15 (0.96–1.38)	1.10 (0.91–1.34)
Women							
Obesity	No	5245	195	98,606	2.0	1.00	1.00
	Yes	1783	102	33,717	3.0	1.44 (1.14–1.83)	1.48 (1.15–1.91)
Elevated blood pressure	No	3657	127	69,337	1.8	1.00	1.00
	Yes	3371	170	63,004	2.7	1.04 (0.82–1.31)	1.02 (0.80–1.31)
Elevated plasma glucose	No	6403	256	120,761	2.1	1.00	1.00
	Yes	625	41	11,556	3.5	1.37 (0.98–1.91)	1.44 (1.03–2.03)
Dyslipidemia	No	5143	208	96,688	2.2	1.00	1.00
	Yes	1885	89	35,664	2.5	1.03 (0.80–1.31)	1.04 (0.80–1.34)

Obesity: body mass index ≥ 25 kg/m^2^

Elevated blood pressure: systolic blood pressure and/or diastolic blood pressure ≥ 130/85 mmHg or the use of antihypertensive medication

Elevated plasma glucose: fasting plasma glucose ≥ 6.1 mmol/L (110 mg/dL) (with a fasting duration of at least 3 h) or casual plasma glucose (for less than 3 h or without regard to the time since the last meal) ≥ 7.8 mmol/L (140 mg/dL) and/or the use of antidiabetic medication

Dyslipidemia: triglycerides ≥ 1.69 mmol/L (150 mg/dL) and/or high-density lipoprotein cholesterol < 1.03 mmol/L (40 mg/dL) and/or the use of antihyperlipidemic medication

*HR* hazard ratio, *CI* confidence interval

^a^Hazard ratios adjusted for age

^b^Hazard ratios adjusted for age, smoking status (never, past, or current smoker), alcohol drinking status (never, past, or current drinker), marital status (yes or no), educational attainment (≤ 15, 16–18, or ≥ 19 years), physical activity (low, middle, high), occupation category (white-collar, blue-collar, or no working), and menopausal status (pre or post) only in women

Table 5 shows HRs and 95% CIs for cancer-type specific mortality with MetS by sex. The multivariate-adjusted HRs of death from colorectal and breast cancers were 3.48 (95% CI 1.68–7.22) and 11.90 (95% CI 2.25–62.84), respectively, in women. However, no significant difference was observed between MetS and any cancer-type specific mortality in men.

**Table 5:** Multivariate analysis of cancer-type specific mortality with metabolic syndrome by sex

	Presence of MetS	Cancer deaths	Parson-years	Crude mortality (/1000 person-years)	HR-age^a^ (95% CI)	HR-all^b^ (95% CI)
Men						
Lung	No	132	2374	1.8	1.00	1.00
	Yes	14	256	1.5	0.91 (0.52–1.58)	1.13 (0.65–1.98)
Stomach	No	54	971	0.76	1.00	1.00
	Yes	9	162	0.96	1.35 (0.67–2.73)	1.29 (0.58–2.88)
Colon and rectum	No	29	521	0.41	1.00	1.00
	Yes	4	72	0.42	1.16 (0.41–3.31)	1.40 (0.48–4.10)
Liver	No	18	324	0.25	1.00	1.00
	Yes	4	72	0.42	1.86 (0.63–5.49)	1.57 (0.45–5.53)
Prostate	No	12	216	0.17	1.00	1.00
	Yes	2	36	0.21	1.56 (0.35–6.99)	1.41 (0.30–6.61)
Women						
Lung	No	30	565	0.25	1.00	1.00
	Yes	6	112	0.52	1.82 (0.76–4.37)	1.66 (0.64–4.31)
Stomach	No	42	791	0.35	1.00	1.00
	Yes	5	94	0.43	1.09 (0.43–2.76)	0.79 (0.24–2.57)
Colon and rectum	No	30	565	0.25	1.00	1.00
	Yes	10	187	0.86	3.07 (1.50–6.27)	3.48 (1.68–7.22)
Liver	No	14	264	0.12	1.00	1.00
	Yes	3	56	0.26	1.97 (0.57–6.84)	2.34 (0.66–8.28)
Breast	No	3	57	0.025	1.00	1.00
	Yes	3	56	0.26	10.70 (2.11–54.36)	11.90 (2.25–62.84)

*MetS* metabolic syndrome, *HR* hazard ratio, *CI* confidence interval

^a^Hazard ratios adjusted for age

^b^Hazard ratios adjusted for age, smoking status (never, past, or current smoker), alcohol drinking status (never, past, or current drinker), marital status (yes or no), educational attainment (≤ 15, 16–18, or ≥ 19 years), physical activity (low, middle, high), occupation category (white-collar, blue-collar, or no working), and menopausal status (pre or post) only in women

Sensitivity analyses performed by excluding participants who were younger than 40 years at baseline were consistent with the primary findings. These analyses are described in Additional file 1: Table S1.

## Discussion

In the present study, we demonstrated that MetS was associated with increased cancer deaths in women, particularly those younger than 65 years, over a mean follow-up duration of 18.5 years. The predictive value for cancer mortality increased with a higher number of MetS components. The results of the present study are important because the predictive value of MetS for cancer mortality in Japan has not been previously proven.

Only four recent cohort studies have reported a relationship between MetS and cancer mortality [14–17]. Prospective cohort studies in the U.S. reported that the National Cholesterol Education Program Adult Treatment Panel III (NCEP-ATP III) MetS using WC was associated with an increased risk of cancer mortality in men [14], or was not divided by sex [15]. Another prospective cohort study in Korea reported that the NECP-ATP III MetS using BMI instead of WC was associated with increased cancer-related mortality in men, but not in women [16]. The participants in the three cited NCEP-ATP III studies were younger than those in the present study. The number of cancer deaths was small and the prevalence of MetS was also low in the younger generation. In addition, while high estrogen levels may protect against the adverse effects of MetS in young women, MetS and central obesity may affect the risk of cancer in postmenopausal women [24–26]. However, the results of sensitivity analyses that excluded participants who were younger than 40 years of age at baseline were similar to the primary findings. The Japan Public Health Center-based prospective study (JPHC), which included 34,051 participants (12,412 men and 21,639 women) over a follow-up of 12.3 years, reported that the Japanese MetS using BMI instead of WC was not associated with significantly increased cancer mortality in either sexes [17]. The reason for these JPHC study results may be that this study calculated BMI using a self-administered questionnaire. The current study corroborates these findings and extends them by demonstrating that MetS predicts cancer mortality in women.

The present study also showed that the linear association between increases in the number of MetS components and cancer deaths, and the pathology of obesity is key in MetS because the presence of obesity affected the relationship between the number of MetS components and cancer mortality, whereas the absence of obesity did not. Previous studies also reported a dose–response relationship between MetS components and cancer mortality [14–16] as well as the risk of cancer [27].

MetS was positively associated with the risk of colorectal and breast cancer deaths in women. However, the number of cancer-type specific deaths was small. Previous cohort studies reported that MetS was positively associated with cancer mortality in the gastrointestinal system [28], particularly that of colorectal cancer [13, 29]. The Japan Collaborative Cohort Study (JACC) of 96,081 participants (40,510 men and 55,571 women), a nationwide prospective cohort study, reported increased colorectal cancer mortality in women, but not men, with diabetes [30]. A previous meta-analysis reported that MetS was associated with the risk of postmenopausal breast cancer [31].

While many definitions of MetS have been used worldwide, such as the NCEP [32, 33] and International Diabetes Federation (IDF) [34], the original MetS diagnostic criteria were defined in Japan [35]. NCEP and IDF representatives recently agreed that obesity is not an essential item for diagnosis because the clustering of metabolic risk factors is more important than obesity [36]. Therefore, only the Japanese criteria for MetS maintains that obesity is an essential component because it plays a major role in MetS [35]. Although the concept requiring obesity as an indispensable item was based on the pathogenesis of MetS, future studies need to focus on identifying the relationship between cancer and MetS using various criteria (see Additional file 1: Table S2).

There was no significant difference in smoking between participants with and without MetS in both sexes. However, men with MetS smoked more ≥ 21 cigarettes per day than men without MetS (not shown in Table, 18.2% vs. 13.1%, P < 0.001). Current smoker in women may have less impact on MetS than in men because women had lower current smoker than men. There was no significant difference in alcohol drinking between participants with and without MetS in men, while women with MetS were significantly lower alcohol drinkers than women without MetS. One of the reasons may be that light to moderate alcohol consumption decreased the incidence of diabetes [37]. The trends in smoking and alcohol drinking of participants with and without MetS were similar in recent Japanese studies [17, 38, 39].

The mechanisms responsible for the relationship between MetS and an increased risk of cancer death remain unclear; however, potential factors include obesity, insulin resistance, and the insulin-like growth factor (IGF) system [40]. Obesity is associated with inflammation, which leads to insulin resistance [41]. Insulin resistance is a key factor in MetS and increases the risk of cancer mortality [42, 43]. Insulin stimulates the synthesis of IGF-1 and leads to tumor growth [44]. The present study demonstrated that MetS increased cancer deaths in women, but not in men. BMI is a useful indicator of overall adiposity, including visceral adipose tissue (VAT), and VAT is more strongly associated with metabolic risk factors in women than in men [45, 46]. VAT has a direct negative effect on health by releasing a larger amount of excess free fatty acids (FFAs) in women than in men [47]. Triglyceride/FFA cycling is central to the obesity-mediated risk of cancer [48]. Further research is needed to confirm the mechanisms underlying sex-related factors.

The present study has several potential limitations. The measurement of MetS was based on a single measurement only at baseline, which made it impossible to evaluate the effect of changes in metabolic risk factors over time on cancer mortality. Furthermore, we defined obesity of MetS using BMI instead of WC. Although WC and BMI can produce slight differences in the diagnostic performance and pathological meaning of MetS, BMI ≥ 25 kg/m^2^ correlates highly well with a WC of ≥ 85 cm in men and ≥ 90 cm in Japan [23]. In addition, owing to the small number of cancer-type specific deaths, there was a possibility of not only beta errors, but also chance.

## Conclusion

The present results suggest that MetS is a significant predictor of cancer death in women. Furthermore, there is a dose–response association between an increasing number of MetS components and cancer mortality. These findings implied that subjects with MetS may need to prevention and management of cancer. Further studies are needed to confirm the influence of MetS on the risk of cancer.

## Additional file

**Additional file 1: Table S1.** Multivariate analysis of cancer mortality with metabolic syndrome by sex, excluding participants <40 years. **Table S2.** Multivariate analysis of cancer mortality with the NCEP-ATP III and IDF by sex. **Figure S1.** The log-negative-log plot of the survival function for the number of metabolic syndrome components against time to death/follow-up time. **Figure S2.** The log-negative-log plot of the survival function for participants with and without metabolic syndrome against time to death/follow-up time.

## Abbreviations

MetS: metabolic syndrome
HR: hazard ratio
CI: confidence interval
CVD: cardiovascular diseases
JMS: Jichi Medical School
BMI: body mass index
PAI: physical activity index
SBP: systolic blood pressure
DBP: diastolic blood pressure
TC: serum total cholesterol
HDL-C: high-density lipoprotein cholesterol
TG: triglyceride
PG: plasma glucose
ICD-10: International Classification of Diseases 10th revision
WC: waist circumference
SD: standard deviation
IQR: interquartile range
NCEP-ATP: National Cholesterol Education Program Adult Treatment Panel
JPHC: the Japan Public Health Center-based prospective study
JACC: the Japan Collaborative Cohort Study
IDF: International Diabetes Federation
IGF: insulin-like growth factor
VAT: visceral adipose tissue
FFAs: free fatty acids
