# Supplementary material for: Metabolic syndrome is a risk factor for cancer mortality in the general Japanese population: the Jichi Medical School Cohort Study
Source: Diabetol Metab Syndr. 2019 Jan 9;11:3. doi: 10.1186/s13098-018-0398-2 (PMC6325756; doi:10.1186/s13098-018-0398-2)
Supplement: Supplementary file 1 — Additional file 1: Table S1. Multivariate analysis of cancer mortality with metabolic syndrome by sex, excluding participants <40 years. Table S2. Multivariate analysis of cancer mortality with the NCEP-ATP III and IDF by sex. Figure S1. The log-negative-log plot of the survival function for the number of metabolic syndrome components against time to death/follow-up time. Figure S2. The log-negative-log plot of the survival function for participants with and without metabolic syndrome against time to death/follow-up time. [file 13098_2018_398_MOESM1_ESM.docx]

Table S1. Multivariate analysis of cancer mortality with metabolic syndrome by sex, excluding participants <40 years

|  | Men |  | Women |  |
| --- | --- | --- | --- | --- |
|  | Without MetS | With MetS | Without MetS | With MetS |
| MetS participants, n (%) | 3,580 (88.2) | 480 (11.8) | 5,843 (90.5) | 613 (9.5) |
| Cancer deaths | 417 | 55 | 248 | 46 |
| Parson-Years | 64,019 | 8,631 | 110,508 | 11,464 |
| Cancer mortality |  |  |  |  |
| Crude mortality (/1,000 person-years) | 6.5 | 6.4 | 2.2 | 4.0 |
| HR-Age^a^ (95% CI) | 1.0 (reference) | 1.11 (0.84–1.47) | 1.0 (reference) | 1.69 (1.23–2.31) |
| HR-All^b^ (95% CI) | 1.0 (reference) | 1.19 (0.89–1.60) | 1.0 (reference) | 1.69 (1.21–2.36) |
| <65 years old |  |  |  |  |
| HR-Age^a^ (95% CI) | 1.0 (reference) | 1.13 (0.80–1.60) | 1.0 (reference) | 1.70 (1.14–2.55) |
| HR-All^b^ (95% CI) | 1.0 (reference) | 1.21 (0.84–1.75) | 1.0 (reference) | 1.66 (1.08–2.55) |
| ≥65 years old |  |  |  |  |
| HR-Age^a^ (95% CI) | 1.0 (reference) | 1.09 (0.68–1.74) | 1.0 (reference) | 1.71 (1.03–2.83) |
| HR-All^b^ (95% CI) | 1.0 (reference) | 1.19 (0.73–1.95) | 1.0 (reference) | 1.69 (0.99–2.89) |

MetS; metabolic syndrome; HR, hazard ratio; CI, confidence interval

^a^ Hazard ratios adjusted for age

^b^ Hazard ratios adjusted for age, smoking status (never, past, or current smoker), alcohol drinking status (never, past, or current drinker), marital status (yes or no), educational attainment (≤15, 16–18, or ≥19 years), physical activity (low, middle, high), occupation category (white-collar, blue-collar, or no working), and menopausal status (pre or post) only in women

Table S2. Multivariate analysis of cancer mortality with the NCEP-ATP III and IDF by sex

|  | Presence | Participants | Cancer deaths | Person-Years | Crude mortality  (/1,000 person-years) | HR-Age^a^ (95% CI) | HR-All^b^ (95% CI) |
| --- | --- | --- | --- | --- | --- | --- | --- |
| Men |  |  |  |  |  |  |  |
| NCEP-ATP III |  |  |  |  |  |  |  |
|  | No | 3,559 | 373 | 64,139 | 5.8 | 1.00 | 1.00 |
|  | Yes | 936 | 100 | 16,723 | 6.0 | 1.12 (0.90–1.40) | 1.19 (0.95–1.50) |
| IDF |  |  |  |  |  |  |  |
|  | No | 3,856 | 410 | 69,218 | 5.9 | 1.00 | 1.00 |
|  | Yes | 639 | 63 | 11,643 | 5.4 | 1.06 (0.81–1.38) | 1.13 (0.85–1.49) |
| Women |  |  |  |  |  |  |  |
| NCEP-ATP III |  |  |  |  |  |  |  |
|  | No | 5,546 | 216 | 104,401 | 2.1 | 1.00 | 1.00 |
|  | Yes | 1,482 | 81 | 27,971 | 2.9 | 1.13 (0.87–1.46) | 1.22 (0.93–1.59) |
| IDF |  |  |  |  |  |  |  |
|  | No | 6,035 | 234 | 113,639 | 2.1 | 1.00 | 1.00 |
|  | Yes | 993 | 63 | 18,733 | 3.4 | 1.44 (1.09–1.91) | 1.52 (1.13–2.03) |

National Cholesterol Education Program ATP III, NCEP-ATP III; International Diabetes Federation, IDF; MetS, metabolic syndrome; HR, hazard ratio; CI, confidence interval

^a^ Hazard ratios adjusted for age

^b^ Hazard ratios adjusted for age, smoking status (never, past, or current smoker), alcohol drinking status (never, past, or current drinker), marital status (yes or no), educational attainment (≤15, 16–18, or ≥19 years), physical activity (low, middle, high), occupation category (white-collar, blue-collar, or no working), and menopausal status (pre or post) only in women

NCEP-ATP III was defined as follows: any three of the following five factors: overweight with a body mass index ≥25 kg/m^2^, elevated blood pressure (BP) as systolic BP and/or diastolic BP ≥130/85 mmHg or the use of antihypertensive medication, elevated plasma glucose (PG) as fasting PG ≥5.5 mmol/L (100 mg/dL) (with a fasting duration of at least 3 hours) or casual PG (for less than 3 hours or without regard to the time since the last meal) ≥7.8 mmol/L (140 mg/dL) and/or the use of antidiabetic medication, hypertriglyceridemia as triglycerides ≥1.69 mmol/L (150 mg/dL) and/or the use of antihyperlipidemic medication, or low high-density lipoprotein cholesterol (HDL-C) as HDL-C <1.03 mmol/L (40 mg/dL) in men and <1.29 mmol/L (50 mg/dL) in women.

IDF was defined as follows: being overweight plus any two of the following three factors: elevated BP, elevated PG, hypertriglyceridemia, or low HDL-C.


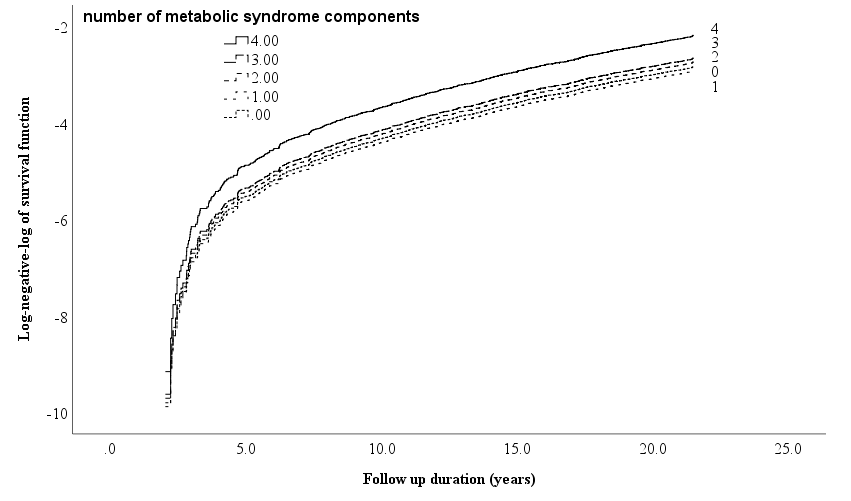


Figure S1. The log-negative-log plot of the survival function for the number of metabolic syndrome components against time to death/follow-up time.


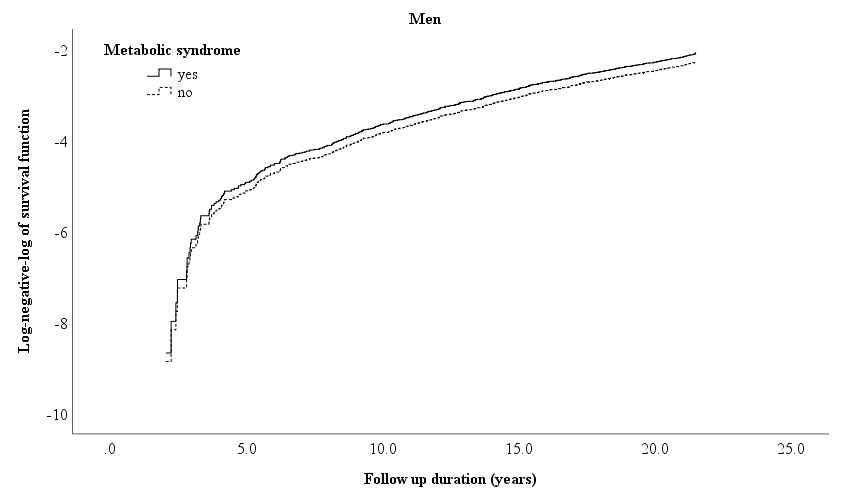


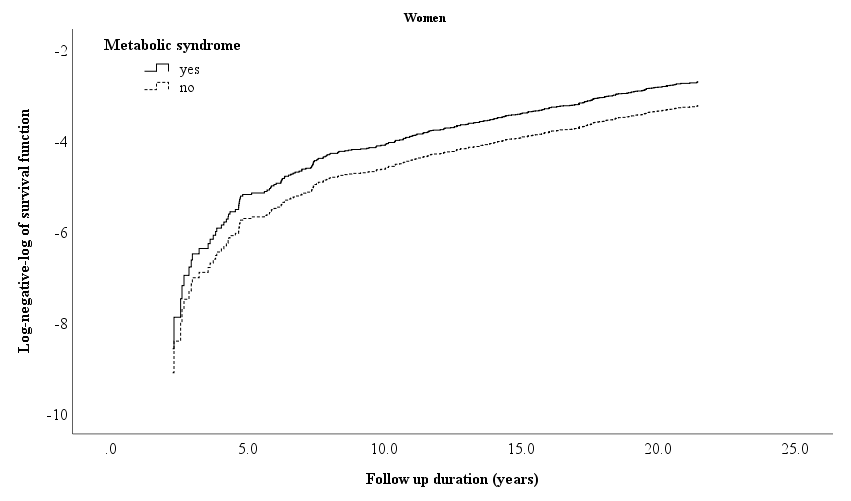


Figure S2. The log-negative-log plot of the survival function for participants with and without metabolic syndrome against time to death/follow-up time.
